# ‘You can give them wings to fly’: a qualitative study on values-based leadership in health care

**DOI:** 10.1186/s12910-019-0374-x

**Published:** 2019-05-27

**Authors:** Yvonne Denier, Lieve Dhaene, Chris Gastmans

**Affiliations:** 1 Department of Public Health and Primary Care, Centre for Biomedical Ethics and Law, Faculty of Medicine, University of Leuven, Leuven, Belgium; 2 Zorgnet-Icuro Care Network Flanders, Brussels, Belgium

**Keywords:** Ethical decision-making, Health care management, Grounded theory, Values-based leadership, Qualitative research, Authentic leadership

## Abstract

**Background:**

Within contemporary health care, many of the decisions affecting the health and well-being of patients are not being made by the clinicians or health professionals, but by those involved in health care management. Existing literature on organizational ethics provides insight into the various structures, processes and strategies - such as mission statement, ethics committees, ethical rounds … - that exist to create an organizational climate, which fosters ethical practices and decision-making It does not, however, show how health care managers experience their job as being intrinsically ethical in itself. In the present article, we investigate the way in which ethical values are present in the lived experiences and daily practice of health care management. What does it imply to take up a managing position within a health care institution and to try to do this in an ethically inspired way?

**Method:**

We carried out a qualitative study (Grounded Theory Approach) to explore the essence of values-based leadership in health care. We interviewed 15 people with extensive experience in health care management in the fields of elderly care, hospital care and mental health care in the various regions of Flanders, Belgium.

**Results:**

Six predominant themes, presented as metaphors, illustrate the essence of values-based leadership in health care management. These are: (1) values-based health care management as managing a large *garden*, (2) as learning and using a foreign *language*, (3) going on a *trekking* with an ethical compass, (4) embodying integrity and authenticity in a credible *encounter* with everyone, (5) being a present and trustworthy leader during *sun and storm*, and (6) contributing to human flourishing by giving people *wings to fly*.

**Conclusions:**

Notwithstanding the importance of organizing a good ethics infrastructure, values-based leadership in health care entails much more than that. It is about the co-creation of an integrated and comprehensive ethical climate of which community-model thinking and authentic leadership are essential components. As a never-ending process, the six metaphors can help leaders to take substantive proactive steps to shape a fruitful ethical climate within their organization.

## Background

During the past two decennia, the focus in health care ethics gradually shifted from the traditional dyadic physician-patient relationship to include emphasis on the decisions made *outside* the clinical setting, which nevertheless significantly *affect* the clinical relationship [1, 2]. The increasing awareness that many of the decisions affecting the health and well-being of patients are not made by the clinicians or health professionals, but by those involved in health care management and policy, gradually raised new and specific ethical questions in contemporary health care [3–7]. Consequently, organizational ethics, also described as “the next step in the evolution of bioethics” [8–10], became an emerging area of importance in health care [11–14].

In its early decades (late 1970s-early 1980s), *bioethics* predominantly focused on ethical issues in the clinical relationship (e.g. patient autonomy, paternalism, truth-telling, ...), in the field of medical research (e.g. human experimentation, power relationships, informed consent, ...) and in the so-called field of Promethean challenges – the powers and responsibilities that came with new knowledge and technologies in medicine and the life sciences (e.g. reproductive technologies, cloning, gene therapy, human genetic engineering, ...) [1]. From the 1990s onwards, the newly emerging field of *organizational ethics*, shifted the focus to ethical issues encountered in management and governance of health organizations, the ethical implications of organizational decision making on key stakeholders (patients, staff and the community); and the ethical complexities of balancing the goal of quality patient care with other important goals such as financial sustainability, staff well-being, learning and innovation, and public accountability’ [11–13, 15–17].

The organizational ethics concern is institutionalized through various formal and informal structures, processes, and strategies [7, 12, 18], such as: mission discernment and value statement [6, 16, 17, 19], ethics committees [20, 21], ethics codes or guidelines, such as codes of conduct or professional codes of ethics [22, 23], ethical rounds, administrative case rounds or ethical roundtables [7, 24, 25], ethics case reflection and moral case deliberation [26–30], ethical decision-making frameworks [15, 31–33], organizational ethics programs [13, 34, 35], accreditation standards and organizational ethics policies [21, 35–37].

### Focus & aim of organizational ethics

Organizational ethics is concerned with the *normative* dimensions of organizational life in health care [4, 10, 16, 17], with ‘the intentional use of *values* in organizational decision-making’ [8], thereby seeking to ‘*define* its core values and mission, *identify* areas in which important values come into conflict, *seek* the best possible resolution of these conflicts, and *manage* its own performance to ensure that it acts in accord with espoused values’ [38]. As a sort of ‘moral compass’, the organizational mission and value statements function as a set of standards according to which the organization’s actions and decisions receive direction and are to be judged [11, 12, 17, 39–41].

The goal of organizational ethics is to realize a strong *alignment* between the stated mission and values and the actual decisions and actions taken by individuals on behalf of the organization [7, 42]. As such, the aim is to create an *organizational climate* that fosters both ethical practices and decision-making [3, 12, 17]. As such, the moral responsibility of a health care organization is conceived as an intrinsic aspect of the *moral identity* of the organization [43].

Since the mid 90’s, many studies were published concerning organizational ethics [11]. Focus of attention are the background, content and nature of organization ethics as a new approach to health care ethics [16, 17, 41], the various forms of ethical infrastructure by which organizational ethics can take shape [7, 12, 18], often in combination with a matrix of strategies and points of action [9, 35, 44–46].

### Organizational ethical frameworks

Among proposed theoretical ethical frameworks for health care services management are the principle-based approach comprising the principles of biomedical ethics: (1) respect for autonomy, (2) beneficence, (3) nonmaleficence, and (4) justice [4, 39] or a combination of the principles with other important ethical goals in health care. Nelson et al. [47, 48] for instance describe the relationship between the ethics principles of autonomy and beneficence with the aim of improving quality and safety in patient care. Other ethical approaches focus on business ethics, managed care ethics and stakeholder perspectives in health care [2, 49–51].

A highly influential ethical framework is that of accountability for reasonableness, developed by Daniels and Sabin [31, 52, 53] paving the way for an ethics of accountable care organizations [44, 54–58]. According to this model, health care organizations engaged in priority setting have a claim to fairness if they comply with four conditions: (1) the rationales for priority setting must be publicly accessible (*publicity condition*), (2) they must be considered by fair-minded people to be relevant to priority setting in that context (*relevance condition*), (3) there must be a way for appealing against these decisions and rationales (*appeals condition*), and (4) there must be some means of guaranteeing that the first three conditions are being met (*enforcement condition*). As such, the ‘accountability for reasonableness’-model provides a *procedural* normative framework by which the fairness of priority setting can be evaluated.

Winkler and Gruen [15] specifically developed a *substantive* normative framework for value-laden decision making within health care organizations, consisting of four substantive principles – (1) provide care with compassion, (2) treat employees with respect, (3) act in a public spirit, and (4) spend resources reasonably – that are derived from the various roles that health care organizations are expected to play as caregivers, employers, citizens and managers. Together, they aim to clarify and resolve existing tensions between the various responsibilities of health care organizations, to promote organizational values and trust in the organization, and to aid discussions about the appropriate roles of health care organizations within the society.

Other theoretical ethical frameworks that guide organizational ethics in a *substantive* normative way are care ethics [59, 60], MacIntyre’s virtue ethics [61–64], and Taylor’s communitarian theory on moral identity [43].

### Organizational ethical issues

From existing empirical research we learn that organizational ethics in health care is concerned with many aspects of organizational life. The most commonly cited ethical issues are [11, 13]: resource allocation, funding, priority setting and distributive justice [14, 32, 65–67]; strategic planning and value setting [68–70]; managing the tension between budgetary constraints and providing high-quality patient care, while trying to safeguard an ethically high standard of clinical care [67, 71, 72], safeguarding justice and access to care, also for the uninsured [73].

Furthermore, there are ethical issues regarding conflict of interest, risk assessment, disclosure of risk and complaints of misconduct, related to issues concerning privacy and confidentiality [74, 75]; agreements over treatment decisions and health services offered [34, 76]. Commonly present are also: guaranteeing the quality of work-life balance and employee satisfaction, workplace ethics and the ethical climate [77–80], trust in organizational leadership and transparency of organizational decision-making [81–85], issues of moral distress among employees that are linked to the organization’s ethical climate [5, 80, 86–90] and of health leaders who are under pressure to justify tough decisions that they do not believe in or feel that are wrong [91].

These examples illustrate how organizational ethical issues essentially fall ‘into three main categories: (1) ethical issues emerging in clinical care because of decisions taken elsewhere in the organization, (2) ethical issues in clinical care with wider-reaching organizational implications, and (3) ethical issues related specifically to the business aspects of healthcare organizations’ [12, 13].

### Lived experiences of health care management

On a more general level, the literature and examples on organizational ethical issues show that health care management decision-makers have to deal with many ethical issues that are at the heart of the contemporary health care debate, viz. that they have to balance cost, access and quality in health care [92]. The basic tension is to preserve a health care system that protects and promotes the best interests of patients and people in need of care, while balancing society’s needs to ensure affordable, accessible and high quality care with finite resources and budget constraints. As such, health care managers are continuously being confronted with the ethical dimensions of key policy, operational and budgetary decisions within their health care organizations.

What this broadly elaborated field of existing literature on organizational ethics does *not show*, however, is how health care managers *experience* the job as *being intrinsically ethical in itself*. What does it mean to be a health care manager in an ethically inspired way? What does it mean to deal with complex ethical issues in an ethically well-founded way? This is the aim and starting point of our study.

## Aim

The research question is: how can all above-mentioned dimensions of organizational ethics be realized in practice? More specifically, what is the essential role of health care management in this regard? As the existing studies indeed illustrate key aspects of organizational ethics, identify essential values and strategies, reveal key ethical dilemmas in health care management, they do not show how this is being realized in practice, as seen through the eyes and *lived experiences* of health care managers [93].

This is an important gap. In the literature, there is much attention for the importance of an *integrated* and *comprehensive* organizational ethics, realizing a strong *alignment* between the mission, vision and values and the actual decisions and actions taken by individuals on behalf of the organization, thereby creating an integrated ethical climate, vouching for organizational *integrity* [7, 12, 17, 42, 43]. Mission statement research has, however, also shown that when all these aspects of organizational ethics are not being supported and truly embodied by the health care leaders and management, it has no impact on reality [19, 94, 95]. Or worse, it can have a negative impact, creating cynicism and perceptions of organizational ethics as a form of window dressing [96].

An important finding in this regard is the strong relationship between the use of ethics and the presence of subjective norm colleagues [19]. It is not so much our objective hierarchical superiors that have a significant impact on our moral sensitivity and ethical behaviour, but our subjective norm colleagues/superiors, i.e. those salient but influential people that indirectly influence our ethical behaviour. Why is that so? Because they are important to us, as a role model. They live up to their values [64, 97–103]. Important for health care managers in this regard is the following question: How can they, next to being objective hierarchical superiors, also function as subjective norm superiors or colleagues? How can they actually realize an integrated organizational ethics framework?

In this study we wanted to find an answer to these questions. What does it actually imply, being an ethically inspired health care manager within a contemporary health care institution? How does he or she experience the practice of leading a health care organization in a values-based way? How can the health care manager make sure that the available ethical infrastructure will have an impact on organizational policy and care practices? How does the health care manager experience the possibilities and limitations of an integrated organizational ethics framework? And what do they do when it becomes tough and really difficult?

## Methods

### Design

As our study aimed to present a rich and in-depth view of health care managers’ ethical experiences and reasoning, we chose a qualitative design, using semi-structured interviews. We selected a grounded theory approach to guide our data collection and analysis [104].

### Participants

We purposefully selected people with extensive experience in health care management (as director and board member) in the fields of elderly care, hospital care and mental health care in the various regions of Flanders, Belgium (factor of homogeneity). In the sampling, we guaranteed heterogeneity of participant characteristics related to age, sex, educational background, work experience, type, size and location of the health care institution they were predominantly affiliated with.

For our study, we explicitly included people with a well-known reputation of practising values-based health care management. They were recognized by many as ethical leaders, even though they never presented themselves as such. The reason we purposefully selected them was twofold. (1) We had broadly-informed sufficient reason to believe that these ethically inspired leaders were going to give us the most in-depth and rich information about their experiences in values-based health care management. And (2), we had good reasons to believe that they were not going to give socially desirable “eager to please” answers to the normative issues that were to be discussed in the interviews.

Participants were included when they met the following inclusion criteria: (1) being employed as health care manager in an elderly care, hospital care, or mental health care institution (or a combination of these institutions within a larger group) in Flanders, Belgium, (2) having at least 3 years of experience in the current position, (3) having a well-known reputation of practising values-based health care management, (4) being Dutch-speaking, and (5) being willing to participate in an in-depth interview about their own experiences and practices of values-based leadership in health care.

The final sample consisted of 15 managers (12 men and 3 women) from not-for-profit health care institutions, geographically spread over the five provinces of Flanders. The participants’ characteristics are summarized in Table 1.

**Table 1:** Characteristics of the Participants (*N* = 15)

	N (%)
Gender	
Female	3 (20)
Male	12 (80)
Age	
40–49	2 (13.3)
50–59	9 (60)
60–69	4 (26.7)
Level of Education	
Advanced Master’s Degree in Medicine	4 (26.7)
Master’s Degree in Economics and Business Administration	5 (33.3)
Master’s Degree in Nursing Science	2 (13.3)
Master’s Degree in Psychology	1 (6.7)
Master’s Degree in Pharmaceutical and Pharmacological Sciences	1 (6.7)
Master’s Degree in Law	1 (6.7)
PhD in Law	1 (6.7)
Additional training and degrees in Health Care Management or Business Administration	15 (100)
Years of Working Experience in Health Care Management	
5–9	2 (13.3)
10–19	5 (33.3)
20–29	3 (20)
30–39	3 (20)
> 40	2 (13.3)
Setting^a^	
Elderly Care	6 (40)
Mental Health Care	4 (26.7)
Hospital Care	10 (66.7)

^a^Multiple items possible: total > 100%

### Data collection

We performed 15 semi-structured one-on-one interviews between March 2012 and September 2013. We asked the participants to describe the way in which ethical values are present in the daily practices of health care management by giving examples of ethical dilemmas in their work, describing situations in which they had to make very hard choices, describing positive and inspiring as well as negative and difficult experiences in health care management. They were also asked to reflect upon people who serve as their own role models of ethical leadership and to describe why exactly these people serve as an ethical example to them. An overview of the main topics from the interview guide is offered in Table 2. The interviews lasted an average of 1.5 h. All the interviews were conducted by one researcher (YD), were audiotaped and transcribed *ad verbatim* with the participants’ consent.

**Table 2:** Interview guide – Main topics

Starting questions	
Can you recall a very difficult ethical dilemma/conflict/contradiction that you have experienced in your professional life as a health care manager?	
Can you describe the situation in detail? What happened exactly?	
Why was it so difficult?	
How did you deal with it?	
Was a solution possible? How did you come to it? If not, what did you do?	
Why was that a striking example of an ethical dilemma to you?	
Ethical Values	
What does ethics mean to you?	
How is it present in your job as health care manager?	
Which values are inspiring to you?	
Which values function as an ethical compass in your job? Why?	
What, according to your experience, does ethics have to do with health care?	
What, according to your experience, are the most difficult or challenging ethical dilemmas in health care management?	
Ethical experience	
Which ethical experience/situation (within you job) has most significantly remained in your memory until now?	
Can you describe it? What happened?	
What exactly made this experience positive/inspiring or negative/difficult?	
Why is it this ethical experience that remained most in your memory?	
Role Model	
Who has been your ethical example?	
Who has been a clear role model in your professional life?	
Can you describe why?	

### Analysis

Data analysis was performed using the Qualitative Analysis Guide of Leuven (QUAGOL), which offers a comprehensive and systematic guide that supports and facilitates the process of qualitative data analysis, according to the grounded theory approach [105]. The QUAGOL process of analysis consists of two parts, each consisting of five stages. The method is systematic and characterized by iterative processes of systematically digging deeper inside the data, constantly moving between the various stages of the process. As such, the QUAGOL aims to stimulate the researchers’ intuition and creativity as optimal as possible.

Interview transcripts were read and discussed by two members of the research team (YD & LD). Dominant themes were identified and coding schemes were developed. By applying the constant comparison method, moving between the coding scheme and the raw data, the research team reached a more general level of abstraction within which the predominant themes became most clear when being understood and presented as general metaphors. Within the research team, we came to the conclusion that we had good reasons for choosing the meta-narrative way of presenting the results via metaphors. Working on the participants’ narratives, we aimed to capture their experiences. We proceeded inductively, identifying segments of the material that constituted units of meaning. Dominant themes were identified and an initial coding scheme was developed, followed by detailed coding of all transcripts. Consequently, emergent themes were clarified. Further discussion and revision was performed by all members of the research team, and independent assessment and interpretation of transcripts by a multidisciplinary team of peers was carried out. Interestingly, we found that the most essential concepts and codes, which described the participants’ experiences of ethical leadership, could be presented as metaphors. That way, the participant’s experiences – which were so rich that a neutral description in terms of concepts or codes would not do justice to their full meaning - could be presented as a meta-narrative (using metaphors), which helped to underline the essence of their experiences more clearly. This was important, since during the discussions, it became clear that a meta-narrative approach, using metaphors, best reflected the richness of our data.

The process of coding and metaphor development was supported by the qualitative software QSR NVivo 8. Results of the analysis were discussed by the full interdisciplinary research team (YD, LD & CG) in order to obtain consensus. Trustworthiness was strengthened by recurrent meetings, discussions and revisions within the research team, by maintaining a decision trail of memos, by confidential discussions with members of the Ethics Committee of Zorgnet-Icuro - Care Network Flanders, and with relevant staff members of the Zorgnet-Icuro Care Network Organization about the research process, selection of participants and preliminary results.

### Ethical considerations

The study was reviewed and approved by the Ethics Committee of Zorgnet-Icuro - Care Network Flanders, an umbrella organization uniting over 775 social profit healthcare organizations in Flanders, Belgium. At the time of the study, the committee was involved in developing a large-scale ethical guideline for values-based health care management. A qualitative study on values-based leadership in health care was necessary to acquire sufficient empirical data regarding values-based health care management and organizational ethics. The aim of this study was to gather in-depth insight into real-life management issues in this regard. Consequently, the results of the study could be incorporated into the large-scale project of developing the ethical guideline. All the members of the Ethics Committee unanimously reviewed and approved the study.

All the participants in the interview study received written (by e-mail) and verbal information about the research project and expected outcome. Each participant provided verbal consent prior to the interview (approved by the Ethics Committee). Since we explicitly and purposefully included people with a well-known reputation (as appointed by their peers) of practising values-based health care management, the participants immediately understood the full aim of our study. As such, prior written and verbal information, was deemed sufficient for informed consent. The verbal information and consent was documented in the *verbatim* transcripts. Participation was voluntary. Participants were free to withdraw from the study at any stage. All data were treated confidentially.

## Results

Which ethical values are present in the daily practice of health care management? What does it imply to take up a leading position within a health care institution and to try to do this in an ethically inspired way? Based on the interviews, we found six dominant themes that illustrate the essence of values-based leadership in health care management as described by the participants. On a more general level of abstraction, these themes become most clear when being presented as a meta-narrative of metaphors. They are: (1) the garden, (2) a foreign language, (3) a trekking, (4) the encounter, (5) sun and storm, and (6) wings to fly. We represent the metaphors in detail below. They are also summarized in Table 3.

**Table 3:** Six Metaphors of Values-Based Health Care Management

The Garden	Refers to the dynamic character of a values-based organisational culture, comparable with managing a large garden with a rich variety of plant life, creating the context for ethics to take place (knowledge, patience, reflection and action, undertaking and resigning, making choices, doing the right thing at the right moment …)
The Scene	
Foreign Language	Just like learning and using a foreign language, the ethical reflection and action has to be practised and used continuously, in all situations and at all levels of the organization. Only thus, the ethical reflection and action can become a habitus, a spontaneous and seemingly automatic, or apparently evident, use of the ethical language by everyone within the organization.
Apparent Evidence	
Trekking	Why does it have to be an ethical language? Why do the values have to be ethical values? Because providing care is not the same as providing a finished product. The care relationship is a journey that is not a planned trip but rather an unpredictable trekking, a joint search for a professional and caring answer to human vulnerability. The ethical compass with ethical values helps us to find the right track.
Why Ethics?	
Credible Encounter	Health care managers create the context for a values-based organizational culture so that everyone involved can do their job in a values-based way. Most importantly in this regard is the way in which directors, managers and board members themselves embody and express certain values (like integrity, authenticity, courage and justice …) in their encounter with everyone involved. They are ethical role models.
Which Values?	
Sun & Storm	This metaphor deals with the specific role and function of health care managers when ethical issues become really hard and difficult. When they feel caught between the devil and the deep blue sea. In such cases, making connection with oneself as a person, with direct colleagues in the management team, with the difficult situation itself by ‘walking around with the bots in the mud’ and by listening to people who serve as a sounding board and critic of the choices that have to be made. Let the sun shine on everyone when things go well, and be a buffer when things get rough.
When it becomes really difficult	
Wings	Inspirational sources for health care managers were: working in a health care organization implies an intrinsic engagement for ethical values and values-based actions; making a difference within society, thinking in large-scale and long-term perspectives; and most importantly, lifting the spirited energy of caregivers and bringing them to a higher level, giving them wings to fly.
What inspires the manager?	

### The garden – *The scene*

On the most fundamental level, we learned from the interviews that the practice of values-based leadership in health care management can best be compared to managing a large garden with a rich variety of plant life, requiring both frequent attention and active maintenance as well as the ability to abide by the natural course of seasons and climate. It involves a combination of being active and passive at the same time. Observing patiently, taking action when necessary, and doing this in a well-balanced way. As such, values-based health care management is about observation, knowledge, reflection, action and the capacity to do the right thing, at the right time, in the right manner. Asking for help or advice when necessary, looking pro-actively to what should be done in the near and further future.

#### Creating the context for a values-based organizational culture

By taking the above-mentioned actions, health care managers create the organizational context within which a values-based culture can take shape, i.e. a culture that is explicitly characterized by particular values and ethical practices.

*I believe that the strength of leadership lies in the fact that you can help create the context within which good examples and particular values come to the front and wrong ones have little opportunity to do so. (Manager hospital care)*

As such, the managers create a context for health professionals so that they can provide values-based care for people in need. Interestingly, most participants referred to person-centred care both for care receivers and care workers as the core value of their managerial work. This implies a dual focus, making sure that both the *patients* receive the care they need and the *care workers* receive the context and means to provide that type of care.

*Everything starts with the patient, actually. That is our final goal. [ … ] As management, it is our job to organize the structure in such a way that we provide optimal, well-organized, professional and qualitative care. Making sure that we provide decent care, good care, values-based care. [ … ] We also have to make sure that the caregivers actually can provide good care, that their working conditions and circumstances are good, and also that they reflect on the given care. That is very important to me. (Manager hospital care)*

Creating a person-centred context both for the care receivers and the care workers also implies, most participants reported, that you have to respect the uniqueness of each individual person. Everyone has a specific personality and particular narrative, a unique life story, and you have to take this into account by asking whether everyone has at one’s disposal what he or she needs in his or her particular position, whether or not you are a care worker or care receiver. Can everyone, patient, health professional, be what he or she wants to be in this particular situation? And even more: are you, as a manager, prepared to make exceptions to existing rules and regulations in order to make sure that everyone *has* what he needs in order to *be* who he or she wants to be?

*Are you prepared to make an exception to existing rules and procedures when you sense ‘Something is wrong. This is not right for this person, we need to make adjustments.’ And yes, are you willing to go far into implementing this idea? (Manager mental health care)*

#### Making values-based choices

Interestingly, all the participants pointed at ‘making choices’ when describing the essence of health care management. Making choices, in a well-balanced combination of facts and values, trying to find a balance between being overly pragmatic on the one hand and idealistically naïve on the other hand, between factual reality and ethical aspiration.

*Pricing, affordability, quality, selective care or general care, these are the dilemma’s that you have to face. These are very concrete issues. How do you deal, for instance, with patients who cannot pay their hospital bills? [ … ] We took the initiative to clear the debts of people who could not afford it. [ … ]. That was a very good initiative, but we also saw the limits of it. When everybody knows about it, then there will be people who can afford to pay the bill, but simply do not want to. [ … ].In the end, efficiency is also an ethical value. When patients do not pay their bills, this is also ethically problematic. When you don’t collect the money, you cannot proceed in providing good care … (Manager in hospital care)*

Within this practice of constantly making choices, setting priorities, trying to find a well-balanced combination of facts and values, all the participants deemed it essentially important to have an ethical framework as benchmark. For in being a guide for tough decisions, these ethical values make difficult choices feasible. The harder the choices, the more important the presence of such an ethical framework, and the presence of dialogue about these values and priorities with the various stakeholders. By way of illustration, we refer to the participant who described a tough case of values-based decision making in the context of major cutbacks:

*Notwithstanding the cutbacks we had to make, it was a good thing to explicitly justify why we continued to organize the care for a particular category of patients, who need a very intensive and costly form of care and for which we do not receive any additional funding. Being able to openly discuss the fact that this is an extremely vulnerable category of patients, that our organization has always taken care for them, and that no one in the near region provides this type of care, fortified our joint decision to continue the care for this most vulnerable group. (Manager in mental health care)*

This case also illustrates that creating a values-based organizational culture is not an individual matter, to be done by the manager on one’s own. It has to be done, together with everyone involved.

*[Our organization] is actually a movement of people that are inspired by the same story [ … ]. The foundational idea has always been: ‘The care we provide can only be as good as the people who provide it’. We cannot write this story by merely composing fascinating regulations or mission statements. No, it has to be written every day, by everyone, in his or her particular position, function or role. (Manager in hospital, elderly and mental health care)*

#### Three dimensions of values-based health care management

All in all, this joint movement implies three dimensions of values-based health care management. The first dimension is that of content (*What do we do?*). It concerns the actual creation of an organizational culture of joint reflection and dialogue about the ethical values that everyone wants to realize in care practice and organization. It implies making sure that the necessary steps to actually *do* something with these ethical reflections are being taken. That the ethical values are really present in reality.

The second dimension is that of method (*How do we do it?)*. How can we realize this? All the participants mentioned that sensitization, education, reflection and discussion regarding ethics has to be made possible within the health care organization and has to be actively supported by the management. As such, the common moral responsibility to provide person-centred care becomes reality and does not stay on the level of formal regulations and mission statements.

The third dimension is that of time and pace (*When do we do it?*) and refers to the fact that one cannot realise an integrated values-based culture by organizing, for instance, one large ethics initiative per year (a conference, an ethics meeting, …). Instead, real ethical reflection has to be part of a continuous process, taking place at frequent moments and various places (going from daily practices and informal moments, to more formal and official meetings on a regular basis) and this on all levels (from boardroom to bedside and back).

To summarize, the garden is the *general metaphor* for a values-based organization. It is the scene where it all happens and within which the management has a specific presence, role and function. The following five metaphors reveal more *specific characteristics* of a values-based organizational culture.

### Foreign language – the apparent evidence of an organizational culture

What exactly is a values-based organizational culture? From the participants’ experiences, we learned that it is best understood as a horizon of meaningful behaviour, norms, values, habits, traditions, codes of conduct etc., which altogether determine the social interactions between people within the organization as well as their various specific ways of dealing with particular situations.

#### Learning by doing

Most participants pointed at the fact that a values-based organizational culture essentially comes down to an active and dynamic culture of reflection and action. There are no pre-given answers to ethical questions. Realizing a values-based organizational culture is a comprehensive process of ‘learning by doing’, a process that affects everyone in the organization, and is not *restricted* to ethics experts, ethics committees, or ethical rounds. For the participants, it was certainly a matter of ‘comprehensive ethics’, understood as:

*A common culture of reflection, weighing matters and values. And it is precisely that reflex of thinking and weighing that you can take up and apply to many dossiers. Also in purely technical dossiers, for instance, where ethical values do not immediately seem to be a main issue, they are nevertheless present. [ … ]. And this also counts for board members, president of the board, for management, head nurses, and so on. All together, we have to pay attention to that ethical dimension. Continuously. Take it up as a full-fledged dimension within our global policy. Integrated and comprehensive. Not separate. (Manager in mental health care)*

#### Apparently evident

All the participants stressed that continuously taking up the ethical dimension of reflection and action - in light of the guiding ethical values - works in a catching and positively contagious way. For then, certain codes of conduct become evident within the organization. They become a ‘habitus’, a normal and natural way of partaking in the organization, in the sense that discussions and decisions within the organization are always being made in the light of the organization’s ethical framework of values and norms. It becomes an integrated part of one’s choices and decisions in health care.

Notwithstanding the fact that the practice of ethical reasoning and action can become a ‘habitus’ within the organization, it nevertheless requires active and regular exercise. For it can only be a habitus when it is actually and consciously done and applied on a regular basis.

*Within an organization, you can do a lot regarding ethics of care. But you can only do this when you have a specific ethical culture and structure within the organization. You can organize ethics by bringing in ethical competence and expertise. By working with senior staff on these matters, by letting yourself be confronted with ethical situations, by bringing it in the group, by actively learning how to deal with vulnerability and dignity, etc … You cannot leave it to the whims of coincidence. You have to be consciously attentive to it. It is not a matter of ‘natural flow’. No, I really believe that you have to work on it, that you have to deal with ethics in a well-considered and professional way. (Manager in elderly care)*

As such, and on a more general level of abstraction, we learn that the best way of illustrating the ethical practice, is by comparing it with *learning and using a foreign language*. In the beginning, it is difficult and bumpy and requires active and explicit exercise. But the more you use it, the more you speak it and apply the correct grammar, the easier it becomes. Until you come to the point that it happens naturally. Then, it has become a habitus, a spontaneous and seemingly automatic use of the language. Nevertheless, you have to continue using it, frequently apply it. Otherwise, you will lose it again. Therefore, it is an apparent ‘habitus’. The ethical discourse is like using a foreign language. In order to apply it in a spontaneous and natural way, you have to use it frequently.

### Trekking – why do we need ethics?

But why, we asked the participants, does this language and guiding framework of values have to be an *ethical* language, or an *ethical* framework? For in making tough decisions or developing fundamental strategies for the organization, one could easily also apply a *rational* or *pragmatic* framework. Why do we need *ethical* values at all? Their answer was straightforward, as one participant told us: “Because it is about care and not about cookies.”

Care touches upon the essence of human vulnerability and dignity, the participants told us. In providing care, we enter into a complex and unpredictable relationship with the care receiver and family. A relationship that starts from the fundamental vulnerability of the care receiver (needs, questions, concerns, worries, fears, emotions …), and within which we try to provide an answer that is person-centred and dignity-enhancing. And within this relationship, we continuously have to search for the right answer to a particular situation. Here, a multidisciplinary group of care providers goes along this journey, together with the care receiver and family. Everyone has a particular assignment, function and role and has to be concerned with the overarching question: “Do we altogether find each other in providing person-centred care for the patient and family?”

*For that which unites us, is the care for the patient. That is our core business. Of course, there are various perspectives of looking at the process, but in the end, it is about us guiding together each particular patient as good as possible through the care process. And everyone involved, has to contribute to this in one way or another. Each from his or her particular function, role and perspective on the case. Sometimes these go together, or overlap. And sometimes they collide. But in the end … when a patient comes to the hospital … It is always a period of anxiety and unease. No matter what happens, it is always compiled with distressing emotions. This implies that the full context of care, being the cleanliness of the room, the quality of the food, the quality of the nursing care, as well as the quality of the medical care, all together have to interact with each other. (Manager in hospital care)*

#### Ethical compass

As such, the managers described, a care relationship cannot be compared with a well-planned linear trip. It is not a finished product (like cookies), that you deliver as the result of a linear service. It is a dynamic relationship, a journey, comparable with a trekking, a backpacking trip. You don’t fully know in advance what lies ahead of you. The possibility of unexpected developments and abrupt deviations, asks for flexibility and adaptability in going along with the care receiver. In continuously trying to find the right track, the participants told us, you need a *compass*. And it is precisely this framework of *ethical* values, that helps us – like a compass - to find the right track. The full quality of the journey is the result of a joint effort. Together trying to find a professional and dignified answer to the particular health needs of the patients and their family. As such, values-based health care management is about creating the right context and structure for care workers so that they can realize values-based care for the care receivers, given the unpredictability of the care process as a whole.

### The credible encounter – which values are we talking about?

A values-based health care organization, most participants told us, can only be managed well, when the managers themselves, by their own attitude and behaviour, embody and promote particular ethical values.

#### Integrity & Authenticity

To all the participants, integrity and authenticity were core values for ethical management. Both values refer to the personal characteristic of honesty and sincerity. Being self-confident and loyal to one’s own personality and values. Being *present* and *attentive,* not staying at a distance. Really ‘be there’ when needed. At the right time, at the right place.

*You have to be there, physically. You cannot run a care organization from an ivory tower, or from Timbuktu by e-mail. Even though it is indeed possible to do so. Once, the grand slogan was: “management by walking around”. Well, that’s the thing. You have to walk around in the house. Best not receive too many files with tables and stuff. All those flashy files from consultants with tables, charts and graphs … I’ve always hated that … No, you have to walk around, listen to the people, keep your finger on the pulse. If you really do this, really master this, you will not make mistakes. But you have to listen really well... and also hear what is left unsaid... (Manager in hospital, elderly and mental health care)*

It also implies *carefulness in decision-making*. Especially when it comes to tough decisions. They may not be taken loosely, but have to be based on legitimate, values-based criteria. One participant described the decision of major collective redundancies:

*The fact that the decision had to be taken, was a clear-cut case. What made it humanly possible for me, to actually do it, was that it was not a blind shot. It was horrifyingly difficult. But the good thing was that I am still, until today, convinced of the fact that this was an ethically justified decision. It was taken very, very careful. Trying to find a balance between business and humanity. What had to be done, had to be done. But it was done in a way that important social and human criteria were taken into account. (Manager in hospital care)*

Authenticity and integrity in management also implies that one really acts according to one’s values; that one represents the values as a person. And precisely this implies taking conscious decisions, embodying consistency and assertiveness, and expressing the *courage* to do the right thing. Really acting on behalf of what one deems important.

*From the moment you sense, in a meeting, that things are going in the wrong direction, you have to have the courage to say this. If it is fundamentally important for the organization and for your own feeling, then you have to be consistent and say: “This is not right”. And I believe that you really have to have the courage to actually do this. Otherwise, you lack authenticity. (Manager in mental health care)*

It also implies the embodiment of *justice*: “Doing justice to everything and everyone.” Making fair and just choices, treating people fairly. According to the interviewees, there is a strong empowerment-idea behind this: providing people (caregivers as well as care receivers) with the real freedom to do and be what they want to do and who they want to be. Providing them with a context within which they can flourish. Values-based health care management implies investing in human flourishing.

#### Walk the talk

Taken together, most interviewees said, integrity and authenticity in management implies that you have to be credible and reliable in all your encounters; to “Walk the talk”, embodying steadiness, honesty and openness in communication and feedback, and being clear and consistent in one’s views and actions. Within the credible encounter, the manager always has to be aware of the fact that he or she functions as a role model:

*How can you, in heaven’s name, ask your care workers to be attentive and caring towards patients, family, colleagues, … to be empathic, … to also bear the psychosocial and spiritual needs of the patients in mind, next to the physical needs, … when you yourself do not show this in your own actions towards them. When they themselves do not feel acknowledged, respected, and valued as a person. I find it incredibly important that the manager functions as a role model. Whether you like it or not, you are being watched, in everything you do. So I think you should try, given the human flaws that we all share, to show what you proclaim in your own behaviour, choices and actions. (Manager in hospital, elderly and mental health care)*

### Sun & Storm – what to do when it becomes really difficult …

Though the concept of ethics and values-based leadership might raise the initial impression that it is all about good things and warm feelings, the participants showed us that management ethics can be very hard, difficult and confronting. The fifth metaphor ‘Sun and Storm’ refers to the fact that values-based leadership is anything but romantic. Several dimensions make it difficult for managers to realize values-based leadership in health care.

#### Unfinished business

First of all, values-based leadership is a continuous process, and therefore it is never finished; it is never fully realized. By that simple fact, the participants told us, it seems like you have never done enough.

*You can never really grasp it. Take, for instance, a building. Today it’s not there, tomorrow it is. Nice. Visible result. Opening. Reception … Whereas continuously putting emphasis on the ethical aspects of care … That is the most difficult challenge. It’s something that requires your continuous attention. And you are never really sure: “Are we really realizing it or not?” (Manager in hospital care)*

#### Between the devil and the deep Blue Sea

Furthermore, the manager’s office is often the place where ethical dilemmas come to the fore and tough decisions have to be made. Given examples of tough decisions were decisions regarding savings, collective discharges because of restructuring projects, having to discharge dysfunctional employees who already face serious personal problems (alcohol, drugs, disease …), having to deal with serious medical or nursing errors … In such cases, all the participants told us, you really feel caught between the devil and the deep blue sea. Moreover, the participants also said that actually, and within a really good values-based organization, it is precisely *only* these very hard cases – “the really tough nuts to crack” - that come to the management’s agenda. All the other decisions should be dealt with at their own level.

*And that is when I say “It’s lonely at the top.” I really appreciate the common work of boards, committees and teams. As long as we ultimately find a solution to a tough problem, we are common owners of the problem, the solution, and the applause … But there are situations where you can’t find a consensus, or solution within the board, committee or team. And then the problem stays with you … leaving your sleep for it … And that’s the loneliness. The fundamental loneliness of being at the top. With all due respect for team work. In such cases … someone has to decide. And people expect it from you. Do not try to escape from it. Because you have to do it. (Manager in hospital, elderly and mental health care)*

#### Making connection

What can you do in such tough cases? All the participants described the importance of making connection, first, with oneself as a person (one’s own talents, capabilities, weaknesses and vulnerabilities) and to share this with the direct colleagues in the management team. Secondly, they referred to the importance of making connection with the particular problem and the persons concerned by gathering sufficient knowledge about the situation and the various decisional possibilities, and by entering into a dialogue with all the relevant persons. This is not easy. You really have to “walk around with your boots in the mud” as one participant described. And thirdly, almost all the participants described the importance of being connected with people who can serve as a sounding board and inspiration when one has to make difficult decisions. They can either be of the same professional background, or do something completely different. What they all shared - these inspirational people - was wisdom and character, patience, ethical engagement, great capability for reflection, experience in respectfully dealing with difficult situations.

*She was a lady I could turn to day and night. With an immense wisdom. And she could say: “I don’t know. Let me think about it. We will speak again in the morning.” How often she said that to me: “I don’t know. Let me think about it.” Understandably … because most things I took to her were … really … Yeah. But she approached these things in a very human way, and always based on human values, never on considerations of profit, or usefulness, or efficiency … I saw that and I thought: “My God … ” (Manager in hospital, elderly and mental health care)*

Also very important, some participants said, is the fact that the manager puts himself in a contradictable position. “As a manager, you have to *want* to be contradicted,” one person said, because it is an essential aspect of the organizations’ values-based functioning. When people are afraid to contradict an idea, hesitant to give their own opinion or to discuss that things are not working right or are not in accordance with the organization’s ethical values, then the values-based character of the organization is empty.

Nevertheless, authenticity and integrity in values-based leadership in health care does not imply that people have to be ethical superheroes. Some participants also mentioned that it is important to be mild to oneself and to other people. Being mild is an essential antidote to perfectionism and hyper idealism. It implies the value of giving ‘a second chance’, being able to deal with human mistakes, together trying to realize the most humanly possible.

#### Après-Vous

Essentially, some interviewees told us, values-based leadership is the kind of leadership where managers stand back when things go well (“During sunny situations”), and celebrate organizational success as the realisation of the whole group. In such cases, the manager gives the floor to the people, letting the sun shine on everyone involved. When things, on the other hand, go wrong, become difficult or really tough (“During storm …” ), then a good leader comes to the front, looks critically at one’s own doings and functions as a buffer for everyone involved.

*Competent leaders are people who look through the window when things go well, and in the mirror when things go wrong. Looking through the window means expressing the ‘après-vous’: bringing the work and effort of others into the light. It isn’t my success. Other people have realized this. On the other hand, when something goes wrong, you have to be there for the people who need you, looking at yourself in the first place, and withholding from accusing other people. Self-reflection and self-critique are very important to me. And in this way, we have to be a role model. To appreciate other people in the first place, and be reflective and critical towards ourselves. (Manager mental health care)*

### Wings – what inspires the manager?

Inspiration and animation are the basic strengths that people need in order to do their job in an engaging way. What inspires the manager of a health care organization? What exactly are the inspirational sources of values-based leadership? First of all, most participants described the fact that working in a health care organization in itself implies an intrinsic engagement for ethical values and values-based actions. Again: “Because it is about care and not about cookies”:

*It [the health care organization] is a place where you, continuously, determine your doings and beings on the basis of what you deem ethically valuable; on the basis of how a society of human beings could be, might be, should be. (Manager elderly care)*

“Making a difference”, was a second important source of inspiration. Step by step making health care better, creating better structures, making it more person-centred, better accessible, improve quality; being able to change structures, improve things, sometimes making the impossible possible; making the world a better place, improving the quality of society, thinking in large-scale and long-term perspectives.

Most importantly, some of the interviewees said, was that as a values-based manager, you have the possibility to lift the spirited energy of people and to bring it to a higher level. “You can give them wings to fly”, one participant said. This was considered to be essentially important. For care workers, they said, are engaged people who act, by nature, in a values-based and person-centred way. They already do this. As a manager - by providing a good structure and context for care workers to realise their ethical engagements - you can contribute even more to this type of human flourishing in caregivers. Moreover, precisely by doing this, by acting and reflecting in a values-based way, ethics can create a boost of positive energy.

*Together dealing with an ethically difficult situation costs a lot of time and energy, but it also creates energy. And that’s what we have to look for, for things that give energy. When you have reflected on something and really considered it from an ethical perspective, I am convinced that as a group, you will derive a lot of positive energy and job satisfaction from the solution that you have found together. These are things that are very important to management and directors: to actually experience this, to become acquainted with the way in which the team handles a very difficult situation, and to congratulate them with the solution. Such things give a boost. To everyone. I am truly convinced that when you act in an ethically inspired way, that it creates a lot of positive energy in the organization. (Manager hospital care)*

## Discussion

### Strengths

#### Substantive complement

This is the first study offering in-depth insight into the *lived experiences* of health care managers of the way in which organizational ethics can be realized in practice. By inquiring into the question which ethical values are present in the daily practice of health care management and what it *actually* means to take up a leading position within a health care institution in an ethically inspired way, our study offers insight into the essential and multidimensional role of health care services management in realizing a values-based organizational culture in health care. As such, it offers a significant and important *substantive* complement to the existing literature on organizational ethics in health care.

#### Clarity by metaphors

By applying the QUAGOL method for data analysis [105], which is characterized by iterative processes of systematically digging deeper inside the data, constantly moving between the various stages of the process and applying the constant comparison method, we reached a high level of abstraction in the results, which allowed us to understand and present the predominant themes as a meta-narrative of metaphors. The advantage of using metaphors is that they clearly present the essential dimensions and associations of the research subject [106], which is, in our case the meaning and content of values-based health care management.

Our study revealed that the essence of values-based leadership in health care management as described by the participants can be understood and presented with the help of six metaphors. These are: (1) values-based health care management as managing a large *garden*, (2) as learning and using a foreign *language*, (3) going on a *trekking* with an ethical compass, (4) embodying integrity and authenticity in a credible *encounter* with everyone, (5) being a present and trustworthy leader during *sun and storm*, and (6) contribute to human flourishing by giving people *wings to fly*. By combining these dimensions, health care managers create the context within which a values-based organizational culture in health care can take place. In this way, the health care manager can actually manage the ethical life of the health care organisation that he or she is responsible for.

#### Creating context for an ethical culture

Like managing a large garden, the practice of values-based health care management requires observation, knowledge, reflection, action and the capacity to do the right thing, at the right time, in the right manner. Asking for help or advice, when necessary, looking pro-actively to what should be done in the near and further future. By doing this, health care managers create the organizational context (the garden, so to say) within which a values-based culture can take shape, i.e., a culture that is explicitly characterized by particular values and ethical practices [11, 42, 86].

As such, we can reach an integrated and comprehensive process: creating context for continuously taking up the ethical dimension of reflection and action, creating an active and dynamic culture of reflection and action, a comprehensive process of ‘learning by doing’, a process that affects everyone in the organization and is not restricted to the formal structures of organizational ethics (ethics experts, ethics committees, ethical rounds, case deliberations …) [107]. By joint reflection and action, speaking a joint ethical language at all the levels of the organization. By consistently using a clear ethical compass [12, 17], even the toughest decisions become feasible, because they are values-based. Interestingly, Brinkley [42] stresses the fact that when ethical culture shaping is done well, “the investment of time, effort and capital will yield dividends for generations to come” (p. 12).

#### Authentic leadership

Our study confirms the essential importance of ethical and authentic leadership. Without authentic leadership, there cannot be a true and steady ethical climate. Authenticity is a psychological concept that reflects knowing, accepting, and acting in accordance with one’s values and beliefs, preferences and emotions [98]. Our results offer an empirical illustration of authentic leadership, understood and described as a “process that draws both from positive psychological capacities and a highly developed organizational context, which results in both greater self-awareness and self-regulated positive behaviours on the part of leaders and associates, fostering positive self-development” (p. 321) [108]. Authentic leaders are seen as people who are hopeful, optimistic, resilient, and transparent. They operate consistently with values that include being present and visible to others, focusing on what is the ethically right thing to do, taking the lead even at personal risk, making the development of others a priority, and working to ensure their communication is transparent and perceived by others as intended.

Our results also confirm the four underlying concepts of authentic leadership, as described by Gardner et al. [99]. These are: (1) *heightened self-awareness* as the process of continually coming to understand one’s unique talents, strengths, sense of purpose, core values, beliefs and wishes. (2) *Balanced processing* as the ability to assess relevant information accurately and from a relatively objective view, consequently acting on these assessments without being diverted by self-protected motives. (3) *Authentic behaviour,* understood as acting in accordance with one’s values and needs (‘Walk the talk’), in order to actually be seen by the colleagues as acting with integrity. (4) *Relational transparency*, involving the presentation of one’s genuine self by being open with regard to one’s values, identity, emotions, and motives. As such, it is a key component of authentic leadership and a significant determinant of trust in the leader [98].

### Limitations

#### Possible bias

As mentioned in the methods section of this paper, we purposefully selected people with extensive experience in health care management. In the sampling, we guaranteed heterogeneity of participant characteristics related to age, sex, educational background, work experience, type, size and location of the health care institution they were predominantly affiliated with. However, we explicitly included people with a well-known reputation of practising values-based health care management. They were experienced by many as ethical leaders, even though they never presented themselves as such. The reason we purposefully selected them was twofold. (1) We had broadly-informed sufficient reason to believe that these ethically inspired leaders were going to give us the most in-depth and rich information about their experiences in values-based health care management. And (2), we had good reasons to believe that they were not going to give socially desirable “eager to please” answers to the normative issues that were to be discussed in the interviews. However, it remains to be born in mind that our participants were people who were generally known to embody ethical leadership.

#### Window dressing

This brings us to the flipside of ethical and authentic leadership, i.e. the issue of window dressing. From the interviews, we learned that values-based health care management is not only about *management of ethics* (by creating the context for organizational ethics to take place). It is also about the *ethics of management* (by embodying integrity and authenticity in one’s own attitude and behaviour). Although we had very good reasons to believe that the participants were not going to give socially desirable answers, there is never 100 % certainty in such cases.

### Organizational ethics culture

The crucial question in this regard is: Is the motivation to ethically inspired health care management and to realize an explicit values-based organizational culture founded on an *intrinsic* ethical inspiration? Because one truly wants to realize the ethical values in practice? Or is it founded on other, extrinsic, and more window dressing-like inspirations? Because one likes to have an ethical image and reputation? Because ethics simply suits well? Because it is a sub-dimension of being a successful manager? Or because it serves our business goals? In the first motivation, ethically founded health care is the goal and end of our actions. In the second motivation, ethics functions as a means to reach another end. A lot depends, here, on the metaphors and models we use to understand the health care organisations themselves. Illuminating in this regard, is the fundamental distinction between the corporate model of health care organisations and the community model [106].

#### Corporate model

Within the corporate model, we equate organizational ethics with corporate health care ethics and look for “an ethical corporate culture that makes ethics as important for health care decisions as clinical data, financial concerns, and legal issues” (p. 8) [8]. Within the corporate model, however, we are primarily concerned with health services management in a business-like model, with money and customer satisfaction. In health care organizations, this translates into a focus on health care as a commodity or product, health care services meeting a market need, and focus on patient satisfaction rather than a primary emphasis on patient care. In health care organisations being corporations, we focus on producer/customer or contractor/client relationships, rather than caregiver/patient relationships. And most importantly, corporations are viewed as primarily self-interested. The extent to which a corporation cares about others is the extent to which caring about others is in the corporation’s best interest. Accordingly, people (whether they are patients, caregivers of supporting staff) are being viewed as important insofar they can help the corporation in meeting its financial goals. This translates in the ethical responsibilities concerning the product and meeting the customer’s desires in as truthful a manner as possible (like informed consent and autonomous decision-making). Of course these are important ethical issues, but the point is that within the corporate or business model, the primary concern of the institution is business, with codes of ethics and values-based management as a means to prevent unethical means of achieving the economic/business goals of the organisation [106]. Here, ethics is one of the means to reach another goal.

#### Community model

The results of our study underpin the community model of health care organizations. When asking after the reasons why the guiding framework of values had to be an ethical framework instead of a rational or pragmatic of even economic framework, the participants answered that it “is about care and not about cookies”. According to them, providing health care services is fundamentally different from manufacturing or offering a safe and finished product of good quality. To them, a health care organization is a community of people, reflecting and discussing while together trying to find a dignified answer to a patient’s situation of vulnerability. As one interviewee said, the central question is: “Do we find each other in providing person-centred care for the patient and family?”

This strongly adheres to the community model to which McCrickerd [106] refers. According to Mc Crickerd, “any collection of individuals characterised as a “community” will be presumed to be populated with people who care about one another, people who prioritise relationships. Associating health care institutions with communities would put relationships between the individuals in the community as well as community wellbeing at the forefront of conversations. […] such a model would also shift hospitals [i.c. health care organisations] away from the presumption that decision be made at the top and then communicated to those the decisions will affect. In the centre of this model are the people and, importantly, all people – from physicians and patients, who are frequent subjects in health care ethics discussions, to other health care professionals and hospital staff who are not.” (p.343) [106].

This, in turn, adheres to the three dimensions of values-based health care management as described by the participants, i.e. (1) the dimension of *content* (the actual creation of an organisational culture of joint reflection and dialogue about the ethical values that we want to realize in the care practises); (2) the dimension of *method* (by stimulating and supporting sensitization, education, reflection and discussion regarding ethics). As such, the common moral responsibility becomes reality and does not stay on the level of formal regulations and mission statements. And (3) the dimension of *time and pace* (realising the ethical reflection as a continuous process, taking place at frequent moments and various places, and this on all levels, from boardroom to bedside).

## Conclusion

Health care managers are continuously being confronted with the ethical dimensions of key policy, operational and budgetary decisions within their health care organizations. This study highlights the way in which they experience their job as being intrinsically ethical in itself.

In addition to the existing literature on the ethics of health care organizations, which predominantly focuses on the presence or absence of ethics infrastructure (such as codes, missions statements, committees, protocols, ethical rounds or case discussions …), our study makes clear that notwithstanding its importance, health care management ethics entails much more than this. The infrastructure is a means (not the end in itself) to support a *comprehensive* ethical culture as a whole.

From the perspective of values-based health care management, two elements turned out to be essential determinants for an integrated and comprehensive ethical culture in health care. The first is that of the *community model*, understanding the health care organization as a moral community of people, all together taking up responsibility and realizing an ethical climate. Values-based health care management plays a crucial role in creating the context for such a community-minded way of thinking (and acting). The second determinant was found in the presence of *authentic leadership*, whereby health care managers deliberately identify and define their own key values, the organization’s key values, and live up to them as examples of the organization’s ethical culture.

As a never-ending process, the *six metaphors* can help leaders to take *substantive* proactive steps to shape and co-create the desired ethical climate within their health care organizations. These metaphors can also be easily used in ethics education concerning ethical leadership as a new and inspiring way of teaching and discussing ethical leadership, *in addition to* and *different from* the various existing tools that are being used in organizational ethics. Exchanging lived experiences of health care managers by way of using metaphors might inspire the minds of ethical leaders much more and deeper than ethics codes, guidelines, mission statements and protocols.

## Data Availability

The raw data generated and/or analysed during the current study are not publicly available due to reasons of privacy and participants’ anonymity. Public release of the data was also not included in the participant consent process. Subject to participant consent, the data are available from the corresponding author on request.

## Abbreviations

QUAGOL: Qualitative Analysis Guide of Leuven

## References

[1] Daniels N (2006). Equity and population health: toward a broader bioethics agenda. Hast Cent Rep.

[2] Weber LJ (2000). Health care management ethics: business ethics with a difference. Bus Ethics Q.

[3] Koskenvuori J, Numminem O, Suhonen R (2019). Ethical climate in nursing environment: a scoping review. Nurs Ethics.

[4] Darr K (2011). Ethics in health services management.

[5] Schluter J, Winch S, Holzhausere K, Henderson A (2008). Nurses’ moral sensitivity and hospital ethical climate: a literature review. Nurs Ethics.

[6] Gallagher JA, Goodstein J (2002). Fulfilling institutional responsibilities in health care: organizational ethics and the role of mission discernment. Bus Ethics Q.

[7] Silverman HJ (2000). Organizational ethics in healthcare organizations: proactively managing the ethical climate to ensure organizational integrity. HEC Forum.

[8] Potter RL (1996). From clinical to organizational ethics: the second stage of the evolution of bioethics. Bioethics Forum.

[9] Potter RL (1999). On our way to integrated bioethics: clinical/organizational/communal. J Clin Ethics.

[10] Bischop LJ, Cherry MN, Darragh M (1999). Organizational ethics and health care: extending bioethics to the institutional arena. Kennedy Inst Ethics J.

[11] Suhonen R, Stolt M, Virtanen H, Leino-Kilpi H (2011). Organizational ethics: a literature review. Nurs Ethics.

[12] Gibson JL, Sibbald R, Connolly E, Singer PA, Singer PA, Viens AM (2008). Organizational ethics. The Cambridge textbook of bioethics.

[13] Gibson JL (2012). Organizational ethics: no longer the elephant in the room. Healthc Manage Forum..

[14] Gibson JL (2007). Organizational ethics and the management of health care organizations. Healthc Manage Forum.

[15] Winkler EC, Gruen RL (2005). Business ethics in health care: beyond compliance.

[16] Hall RT (2000). An introduction into health care organizational ethics.

[17] Spencer EM, Mills AE, Rorty MV, Werhane PH (2000). Organization ethics in health care.

[18] Rorty MV (2000). Ethics and economics in healthcare: the role of organization ethics. HEC Forum.

[19] Desmidt S, Prinzie A, Heene A (2008). The level and determinants of mission statement use: a questionnaire survey. Int J Nurs Stud.

[20] Marcus BS, Shank G, Carlson JN, Venkat A (2015). Qualitative analysis of healthcare professionals’ viewpoints on the role of ethics committees and hospitals in the resolution of clinical ethical dilemma’s. HEC Forum.

[21] Frolic AN, Drolet K (2013). Ethics policy review: a case study in quality improvement. J Med Ethics.

[22] Yallop AC (2012). The use and effectiveness of codes of ethics – a literature review. Marketing from information to decision marketing from information to decision; Cluj.

[23] Hoeyer K, Lynoë N (2009). An organizational perspective on ethics as a form of regulation. Med Health C and Philos.

[24] Silén M, Ramklint M, Hansson MG, Haglund K (2016). Ethics rounds: an appreciated form of ethics support. Nurs Ethics.

[25] Baumrucker SJ, Schmidt LS, Stolick M, Adkins RW, Carter GT, Oertli KA (2013). Ethics roundtable: can health care mandate drug rehabilitation as a precondition for treatment?. Home Health Care Manag Pract.

[26] Rasoal D, Skovdahl K, Gifford M, Kihlgren A (2017). Clinical ethics support for healthcare personnel: an integrated literature review. HEC Forum.

[27] Stolper M, Molewijk B, Widdershoven G (2015). Learning by doing. Training health professionals to become facilitator of moral case deliberation. HEC Forum.

[28] Stolper M, Molewijk B, Widdershoven G. Bioethics education in clinical settings: theory and practice of the dilemma method of moral case deliberation. BMC Med Ethics. 2016;17(1):45. 10.1186/s12910-016-0125-1 PMC4957934 27448597

[29] Dauwerse L, Abma T, Molewijk B, Widdershoven G (2011). Needs for ethics support in healthcare institutions: view of Dutch board members and ethics support staff. J Med Ethics.

[30] Molewijk B, Abma T, Stolper M, Widdershoven G (2008). Teaching ethics in the clinic. The theory and practice of moral case deliberation. J Med Ethics.

[31] Daniels N, Sabin JE (2002). Setting limits fairly: can we learn to share medical resources?.

[32] Gibson JL, Martin DK, Singer PA (2005). Evidence, economics, and ethics: resource allocation in health services organizations. Healthc Q.

[33] Martin DK, Shulman K, S-S P, Singer PA (2003). Priority setting and hospital strategic planning: a qualitative case study. J Health Serv Res Policy.

[34] Sabin JE, Cochran D (2007). Confronting trade-offs in health care: Harvard pilgrim health Care’s organizational ethics program. Health Aff.

[35] Mills AE, Spencer EM (2005). Values based decision making: a tool for achieving the goals of health care. HEC Forum.

[36] JCI Joint Commission International (2018). Accreditation standards for hospitals (6th ed.).

[37] JCAHO Joint Commission for Accreditation of Health Care Organizations (2019). Comprehensive Accreditation Manuals (annually updated).

[38] Pearson SD, Sabin JE, Emanuel E (2003). No margin, no mission: health care organizations and the quest for ethical excellence.

[39] Donnellan J (2018). Achieve organizational integrity. Aligning ethical principles with health care leadership. Health C Manage Eth.

[40] Iltis S (2005). Values based decision making: organizational mission and integrity. HEC Forum.

[41] Boyle PJ, DuBose ER, Ellingson SJ, Guinne DE, McCurdy DB (2001). Organizational ethics in health care: principles, cases and practical solutions.

[42] Brinkley RW (2013). The case for values as a basis for organizational culture. Front Health Serv Manag.

[43] Pijnenburg M, Gordijn B (2005). Identity and moral responsibility of healthcare organizations. Theoret Med Bioeth.

[44] DeCamp M, Farber NJ, Torke AM, George M, Berger Z, Keirns CC (2014). Ethical challenges for accountable care organizations: a structured review. J Gen Int Med.

[45] Murphy T, Fillatre T (2009). Reflections on health care leadership ethics. Healthc Manage Forum.

[46] Goodstein J, Potter RL (1999). Beyond financial incentives: organizational ethics and organizational integrity. HEC Forum.

[47] Nelson WA, Gardent PB, Shulman E, Splaine ME (2010). Preventing ethics conflicts and improving healthcare quality through system redesign. Qual Saf Health Care.

[48] Nelson WA (2011). Ethics: A foundation for quality. Healthc Exec.

[49] McLeod MS, Tyge Paine G, Evert RE (2016). Organizational ethics research: a systematic review of methods and analytical techniques. J Bus Ethics.

[50] Gilmartin MJ, Freedom ER (2002). Business ethics and health care: a stakeholder perspective. Health Care Manag Rev.

[51] Weber LJ (2001). Business Ethics in Health Care. Beyond Compliance.

[52] Daniels N, Sabin JE (1997). Limits to health care: fair procedures, democratic deliberation and the legitimacy problem for insurers. Philos Public Aff.

[53] Daniels N, Sabin JE (1998). Ethics of accountability in managed care reform. Health Aff.

[54] Lewis VA, D’Annuno T, Murray GF, Shortell SM, Colla CH (2018). The hidden role the management partners play in accountable care organizations. Health Aff.

[55] Gibson JL, Martin DK, Singer PA. Setting priorities in health care organizations: criteria, processes, and parameters of success. BMC Health Serv Res. 2004;4(1):25. 10.1186/1472-6963-4-25 PMC518972 15355544

[56] Martin DK, Giacomini M, Singer PA (2002). Fairness, accountability for reasonableness, and the views of priority setting decision-makers. Health Policy.

[57] Daniels N (1999). Decisions about access to health care and accountability for reasonableness. J Urban Health.

[58] Daniels N (2000). Accountability for reasonableness. BMJ..

[59] Hawk T, Hamington M, Sander-Staudt M (2011). An ethics of care: a relational ethics for the relational characteristics of organizations. Applying care ethics to business.

[60] Hamington M, Sander-Staudt M (2011). Applying care ethics to business.

[61] Lindebaum D, Geddes D, Gabriel Y (2017). Moral emotions and ethics in organisations: introduction to the special issue. J Bus Ethics.

[62] Nielsen RP (2006). Introduction to the special issue: in search of organizational virtue: moral agency in organizations. Organ Stud.

[63] Beadle R, Moore G (2006). MacIntyre on virtue and organization. Organ Stud.

[64] Weaver GR, Treviňo LK, Agle B (2005). “Somebody I look up to.” Ethical role models in organizations. Organ Dyn.

[65] Bosa IM (2010). Ethical budgets: a critical success factor in implementing new public management accountability in health care. Health Serv Manag Res.

[66] Foglia MB, Pearlman RA, Bottrell MM, Altemose JA, Fox E (2007). Priority setting and the ethics of resource allocation in VA healthcare facilities: results of a survey. Organ Ethic.

[67] Cooper RW, Frank GL, Gouty CA, Hansen MC (2002). Key ethical issues encountered in healthcare organizations: perceptions of nurse executives. J Nurs Adm.

[68] Rocker GM, Cook DJ, Martin DK, Singer PA (2003). Seasonal bed closures in an intensive care unit: a qualitative study. J Crit Care.

[69] Arnold L, Drenkard K, Ela S, Goedken J, Hamilton C, Harris C (2006). Strategic positioning for nursing excellence in health systems: insights from chief nursing executives. Nurs Adm Q.

[70] Carney M (2006). Positive and negative outcomes from values and beliefs held by healthcare clinician and non-clinician managers. J Adv Nurs.

[71] Torjuul K, Sorlie V (2006). Nursing is different than medicine: ethical difficulties in the process of care in surgical units. J Adv Nurs.

[72] Kälvemark Sporrong S, Arnetz B, Hansson MG, Westerholm P, Höglund AT (2007). Developing ethical competence in health care organizations. Nurs Ethics.

[73] Gallego G, Taylor SJ, Brien JA (2009). Funding and access to high cost medicines in public hospitals in Australia: decision-makers’ perspectives. Health Policy..

[74] Shannon SE, Foglia MB, Hardy M, Gallagher TH (2009). Disclosing errors to patients: perspectives of registered nurses. Int Comm J Qual Patient Saf.

[75] Jurkiewicz CL, Thompson CR (2000). Conflict of interest: organizational vs. executive ethics in health care. J Health Hum Serv Adm.

[76] Higgins W (2000). Ethical guidance in the era of managed care: an analysis of the American College of Healthcare Executives’ code of ethics. J Healthcare Manag.

[77] Ulrich CM, Soeken KL, Miller N (2003). Ethical conflict associated with managed care: views of nurse practitioners. Nurs Res.

[78] Shirley MR (2005). Ethical climate in nursing practice: the leader’s role. JONAS Healthc Law Ethics Regul.

[79] Rathert C, Fleming DA (2008). Hospital ethical climate and teamwork in acute care: the moderating role of leaders. Health Care Manag Rev.

[80] Bell J, Breslin JM (2008). Healthcare provider moral distress as a leadership challenge. JONAS Healthc Law Ethics Regul.

[81] Hart SE (2005). Hospital ethical climates and registered nurses’ turnover intentions.

[82] Wall S (2007). Organizational ethics, change, and stakeholder involvement. HEC Forum.

[83] Gibson Jennifer, Mitton Craig, DuBois-Wing Gwen (2011). Priority Setting in Ontario's LHINs: Ethics and Economics in Action. Healthcare Quarterly.

[84] Hall LM, Doran D (2007). Nurses’ perceptions of hospital work environments. J Nurs Manag.

[85] Gelsema TI, Van Der Doef M, Maes S, Janssen M, Akerboom S, Verhoeven C (2006). A longitudinal study of job stress in the nursing profession: causes and consequences. J Nurs Manag.

[86] Bachman B (2017). Ethical leadership in organizations: concepts and implementation.

[87] Oh Y, Gastmans C (2015). Moral distress experienced by nurses: a quantitative literature review. Nurs Ethics.

[88] McCarthyy J, Gastmans C (2015). Moral distress: a review of the argument-based nursing ethics literature. Nurs Ethics.

[89] Sutinen R, Kivimäki M, Elovainio M, Virtanen M (2002). Organizational fairness and psychological distress in hospital physicians. Scand J Public Health.

[90] Kivimäki M, Elovainio M, Vahtera J, Ferrie J, Theorell T (2003). Organizational justice and health of employees: prospective cohort study. Occup Environ Med.

[91] Mitton C, Peacock S, Storch J, Smith N, Cornelissen E (2011). Moral distress among health system managers: exploratory research in two British Columbia health authorities. Health Care Anal.

[92] Denier Y (2007). Efficiency, justice and care. Philosophical reflections on scarcity in health care.

[93] Dierckx de Casterlé B, Verhaeghe S, Kars M, Coolbrandt A, Stevens M, Stubbe M (2011). Researching lived experience in health care: significance for care ethics. Nurs Ethics.

[94] Vandijck D, Desmidt S, Buelens M (2007). Relevance of mission statements in Flemish not-for-profit healthcare organizations. J Nurs Manage.

[95] Desmidt S, Heene A (2007). Mission statement perception: are we all on the same wavelength? A case study in a Flemish hospital. Health Care Manag Rev.

[96] Parsons PJ (2008). Ethics in public relations. A guide to best practice.

[97] Mihelič KK, Lipičnik B, Tekavčič M (2010). Ethical Leadership. Int J Manag Inf Syst.

[98] Wong CA, Cummings GG (2009). The influence of authentic leadership behaviors on trust and work outcomes of health care staff. J Leadersh Stud.

[99] Gardner WL, Avolio BJ, Luthans F, May DR, Walumba F (2005). “Can you see the real me?” a self-based model of authentic leaders and follower development. Leadership Quart.

[100] Stander FW, de Beer LT, Stander MW. Authentic leadership as a source of optimism, trust in the organization and work engagement in the public health sector. SA J Hum Resour Manag. 2015;13(1):1–12.

[101] Jordan J, Brown ME, Treviňo LK, Finkelstein S (2013). Someone to look up to: executive-follower ethical reasoning and perceptions of ethical leadership. J Manag.

[102] Storr L (2004). Leading with integrity: a qualitative research study. J Health Organ Manag.

[103] Frisch C, Huppenbauer M (2014). New insights into ethical leadership: a qualitative investigation of the experiences of executive ethical leaders. J Bus Ethics.

[104] Corbin J, Strauss A (2015). Basics of qualitative research. Techniques and procedures for developing grounded theory.

[105] Dierckx de Casterlé B, Gastmans C, Bryon E, Denier Y (2012). QUAGOL: a guide for qualitative data analysis. Int J Nurs Stud.

[106] McCrickerd J (2000). Metaphors, models and organisational ethics in health care. J Med Ethics.

[107] Reiser SJ (1994). The ethical life of health care organizations. Hast Cent Rep.

[108] Avolio BJ, Gardner WL (2005). Authentic leadership development: getting to the root of positive forms of leadership. Leadership Quart.

